# Influence of inflammasome *NLRP3*, and *IL1B* and *IL2* gene polymorphisms in periodontitis susceptibility

**DOI:** 10.1371/journal.pone.0227905

**Published:** 2020-01-24

**Authors:** Josiane Bazzo de Alencar, Joana Maira Valentini Zacarias, Patrícia Yumeko Tsuneto, Victor Hugo de Souza, Cléverson de Oliveira e Silva, Jeane Eliete Laguila Visentainer, Ana Maria Sell

**Affiliations:** 1 Department of Clinical Analysis and Biomedicine, Post-Graduation Program in Biosciences and Physiophatology, State University of Maringá, Maringá, Paraná, Brazil; 2 Department of Dentistry, State University of Maringá, Maringá, Paraná, Brazil; 3 Department of Basic Health Sciences, State University of Maringá, Maringá, Paraná, Brazil; University of the Pacific, UNITED STATES

## Abstract

The pathogenesis of periodontitis (PD) involves several molecules of the immune system that interact in a network to eliminate the periodontopathogens, yet, they contribute to periodontal tissue destruction. The different mechanisms that lead to periodontal tissue damage are not clear. Despite this, immune response genes have been related to the development of PD previously, such as those involved in inflammasomes which are multiprotein complexes and cytokines including Interleukin-1. The aim of the study was to evaluate the polymorphisms in *NLRP3* inflammasome, cytokine and receptor of cytokines genes in the development of periodontitis. This case-control study was conducted in 186 patients with PD (stage II and III and grade B) and 208 controls (localized gingivitis and periodontally healthy individuals). Genotyping was performed using PCR-RFLP for the SNP rs4612666 in *NLRP3* and using PCR-SSP for *IL1A*, *IL1B*, *IL1R*, *IL1RN*, *IL4RA*, *INFG*, *TGFB1*, *TNF*, *IL2*, *IL4*, *IL6*, and *IL10*. Cytokine serum levels were measured using Luminex technology. SNPStats and OpenEpi software were used to perform statistical analysis. The higher frequencies of *NLRP3* T/C and *IL1B* -511 T/T genotypes and *IL2* (+166, -330) GT haplotype were observed in patients with PD compared to controls. The SNPs in *NLRP3*, *IL1R* +1970, *IL6*–174, *TNF* -308, *IL2* +166 and -330, *TGFB1* +869 and +915, *IL4RA* +1902, *IL4*–1098 and -590 were associated to PD in men. In conclusion, polymorphisms in *NLRP3*, *IL1B* and *IL2* genes were associated to PD susceptibility. Men carrying the *NLRP3*, *IL1R*, *IL6*, *TNF*, *IL2*, *TGFB1*, *IL4RA* and *IL4* polymorphisms had greater susceptibility than women for developing PD.

## Introduction

Periodontitis (PD) is a common infectious disease in the oral cavity, affecting about 20–50% of the population in the world [1,2]. The disease initiates with a bacterial invasion in the periodontal tissue which induces the activation of immune response [3] and, the persistence of pathogens and the imbalance in the host immune response, lead to progressive periodontium tissue damage [4,5]. In addition, genetic and epigenetic factors contribute to the development of PD such as individual differences in the host immune response, smoking habits, gender, poor oral-hygiene, and systemic diseases as diabetes mellitus and rheumatic diseases [1]. Genetic variants that influence the susceptibility and the severity of periodontitis arise from changes that occur in the genes and in the biological molecules that they encode [6,7] including cytokines [8–13].

Cytokines are soluble mediators produced by resident cells (epithelial and fibroblasts) and phagocytes in the early chronic phases of PD inflammation, and by T and B lymphocytes in established and advanced lesions in the periodontium [14]. However, the unbalanced production of pro and anti-inflammatory cytokines induces severe damage in the periodontal tissue [15]. Interleukin (IL)-1, IL-8 and tumor necrosis factor (TNF)-α, produced by fibroblasts, promote neutrophils chemotaxis in the inflamed periodontal site. IL-1 can also enhance the expression of the receptor-activator of nuclear factor-kappa B (NF-κB) ligand (RANKL) on osteoblasts. RANKL is an osteoclastogenic factor that upregulates alveolar bone loss. TNF-α in synergism with IL-6 promotes osteoclast differentiation and IL-6 can stimulate the stromal cells to produce RANKL. Thus, these cytokines also promote bone resorption in PD [16]. Usually these proinflammatory cytokines increase in the gingival crevicular fluid (GCF) of PD individuals compared to those without PD [17]. In contrast, IL-4 and IL-10 have supressive properties and can attenuate the tissue distruction in PD. Nevertheless, they were found in lower concentrations in the biological fluids of PD patients [18].

Among the cytokines involved in the pathogenesis of PD, IL-1β, an inflammatory cytokine, can be highlighted for its contribution in stimulating the recruitment and differentiation of osteoclasts in the tissues. Thus, IL-1β contributes to bone resorption in PD. IL-1β levels were higher in the serum, GCF, saliva and gingival tissue of PD patients, and this cytokine could be a potential marker in the management of the disease [19,20]. The decreased levels of this cytokine were found in the GCF after non-surgical periodontal therapy [21–23], but not in all cases [24,25]. Thus, other pathways related to host immune response modulation may be influencing the maintenance of IL-1β levels in the periodontal tissue.

The maturation of IL-1β and its subsequent secretion are dependent on an oligomeric assembly of multiprotein complex called inflammasome. Inflammasome complex consists of cytosolic pattern recognition receptors (PRRs), apoptosis-associated speck-like protein containing a caspase activation and recruitment domain (ASC) and pro-caspase-1 [26]. PRRs such as nucleotide-binding and oligomerization domain (NOD)-like receptors (NLRs) and absent in melanoma 2 (AIM2)-like receptors (ALRs) are activated by pathogen-associated molecular patterns (PAMPs) or danger-associated molecular patterns (DAMPs). Upon sensing the stimuli, the pro-caspase-1 is activated to cleave the IL-1β into its bioactive form. Several inflammasomes have been described: NLR family pyrin domain-containing 1 (NLRP1), NLRP2, NLRP3, NLR family CARD domain-containing 4 (NLRC4) and AIM2 [27]. NLRP3 is the better characterized member and shown to be involved in the innate immune reaction of infectious, inflammatory and chronic diseases [28,29]. Overexpression of NLRP3 in the gingival tissue and increased salivary levels of NLRP3 were observed in PD patients [30]. Upregulation of the inflammasome may lead to an increase in IL-1β production [31,32]. Some therapeutic pathways, based on inhibition of the NLRP3 inflammasome, have been effective in the treatment of experimental diabetic periodontitis, inflammatory diseases and osteoarthritis [33–35]. NLRP1, NLRP2, NLRC4 and AIM2 were also evaluated in PD, however the results about their expression in periodontal tissue were controversial [31,32,36–40].

IL-2 also stimulated the osteoclast activity, contributing to bone resorption in the PD [41]. IL-2 is a proinflammatory cytokine involved in the cell-mediated immunity. It is produced mainly by T helper (Th)-1 cell and promotes the activation, growth and differentiation of T cell subsets, B lymphocytes and natural killer (NK) cells [42]. The ratios for IL-2 and IL-1 and for IL-2 and IL-17A had higher values in PD patients than in individuals without the disease, demonstrating potential for being a diagnostic biomarker [43].

Although some immune genetic variants have been associated with periodontitis, the immunopathogenesis of this disease has not yet been fully understood. Added to this, different ethnic groups may have varying degrees of susceptibility to the disease due to the influence of genetic polymorphisms [44]. No association studies between PD and single nucleotide polymorphisms (SNPs) in inflammasome, in *IL1*, *IL4*, *IFNG*, *TGFB*, *TNF*, *IL2*, *IL4*, *IL6*, *IL10*, *IL1R* and *IL4RA* genes were performed in a population from southern of Brazil. Thus, the aim of this study was to investigate the influence of polymorphisms in inflammatory mediator’s genes in the immunopathogenesis of PD in a population from southern of Brazil.

## Materials and methods

### Sample selection

This case control study included a total of 394 individuals (case/control: 186/208) selected in dentistry clinics from the State University of Maringá and Uningá University Center, between 2012 and 2018. The selection criteria was defined according to the International Workshop for a Classification of Periodontal Diseases and Conditions of 1999 [45]. The included clinical parameters were analyzed at four sites (mesial, vestibular, distal and lingual) of each tooth: probing depth (PD), bleeding on probing (BOP) and clinical attachment level (CAL). The case group was composed of individuals who had at least five sites in different teeth with PD ≥ 5mm, CAL ≥ 3mm and more than 25% of BOP; the control group was consisted of individuals with no pocket ≥ 4mm and less than 25% of BOP. Among all the patients included in this study, eighty-two patients were classified according to PD extent and to PD severity. Of these, 30 patients and 8 controls, all nonsmokers and matched by gender and age, had serum samples obtained for cytokine measurements. According to the classification on periodontal diseases of 2017 [46], the patients can be included in the following categories: stage II and III (based on severity, complexity, extension and distribution) and grade B (moderate rate of progression); and the controls can be consisted of individuals with localized gingivitis or with periodontal health.

The studied population was from North and Northwestern Paraná (22°29'30"-26°42'59"S and 48°02'24"-54°37'38"W), Southern Brazil, over 30 years of age and with at least twenty teeth in their oral cavity. Ethnically, they were classified as a mixed population, due to the great miscegenation process occurred in the state of Paraná and according to previous classifications [47,48]. Individuals with aggressive PD (stage IV and grade C of periodontitis), diabetes mellitus, acute infections, rheumatic diseases, gingivitis, and pregnant women were not included. This study was approved by the Human Research Ethics Committee of the State University of Maringá (COPEP-UEM—No. 719/2011, 02/12/2011 and 1.866.509, 14/12/2016).

Regarding smoking habits, all individuals were classified as smokers and nonsmokers. Information on the patient’s smoking history was obtained through anamnesis. People who had quit smoking for less than 10 years and those who did not remember how long they had stopped smoking were included in the smoking group [49].

### Sample collection

The peripheral blood was collected from individuals and maintained in sterile tubes with EDTA and clot activator for further processing. The buffy coat was obtained and stored inside cryopreservation tubes at -20°C for future analysis and the serum samples were kept at -80°C until analysis.

### DNA extraction and genotyping

DNA was extracted from buffy coat using salting out method [50]. Genotyping for *NLRP3* gene, rs4612666, was performed only in nonsmokers using polymerase chain reaction-restriction fragment length polymorphism (PCR-RFLP). The primer sequences were based on what was previously described [51] and tested on Primer Blast (NCBI) (https://blast.ncbi.nlm.nih.gov/Blast.cgi). PCR mixture was performed in a total volume of 15 μL containing 50 ng of DNA, 1.2 ng/μL from each primer, 0.12 mM of dNTP, PCR buffer, 1.5 mM of MgCl_2_ and 0.5 U of Taq DNA polymerase (Invitrogen Life Technologies, Grand Island, NY, USA). PCR products were digested by *Bpi*I restriction enzyme (Anza^™^, Invitrogen, Life Technologies, Grand Island, NY) at 37°C for 3 hours, and digestion products were visualized on a 2% agarose gel. The quality of genotyping was controlled using internal control for SNP variant in the reactions and by direct sequencing. SBT was performed using two samples for each variant, sequencing with the *BigDye*^*™*^ terminator v3.1 cycle sequencing kit (Applied Biosystems, Thermo Fisher Scientific, USA) according to manufacturer´s instructions on an automatic analyzer (Applied Biosystems 3500xL).

Genotyping of cytokine polymorphisms was performed for smokers and nonsmokers. The following polymorphisms were evaluated by polymerase chain reaction with sequence-specific primers (PCR-SSP) using the Invitrogen kit Cytokines^®^ (Canoga Park, USA): *IL1A* -889 C>T (rs1800587); *IL1B* -511 C>T (rs16944), +3962 C>T (rs1143634); *IL1R* C Pst-1 +1970 C>T (rs2234650); *IL1RN* mspa-1 11100 T>C (rs315952); *IL4RA* +1902 G>A (rs1801275); *INFG* UTR 5644 A>T (rs2430561); *TGFB1* +869 T>C (rs1982073), +915 G>C (rs1800471); *TNF* -308 G>A (rs1800629), -238 G>A (rs361525); *IL2–*330 T>G (rs2069762), +166 G>T (rs2069763); *IL4–*1098 T>G (rs22432484), -590 C>T (rs2243250) and -33 C>T (rs2070874); *IL6–*174 G>C (rs1800795), -597 nt565 G>A (rs1800797); and *IL10–*1082 A>G (rs1800896), -819 C>T (rs1800871), -592 C>A (rs1800872). The PCR products were analyzed by electrophoresis on a 3% agarose gel with SYBR^™^ Safe (Invitrogen Life Technologies, Grand Island, NY, USA) and visualized under UV light. Non-pairing of samples in all the SNPs studied was due to the lack of some samples in the course of the study.

### Determination of the cytokine’s serum concentration

IL-1β, IL-8, TNF-α, IL-6, IL-2, IL-5, interferon (IFN)-γ, IL-4, IL-10 and granulocyte-macrophage colony-stimulating factor (GM-CSF) were quantified in the serum of 30 patients and 8 controls using Luminex technology with the human cytokine 10-plex panel (Invitrogen, ThermoFisher Scientific, Inc., Burlington, Ontario, Canada) in accordance with the manufacturer’s instructions. Samples were diluted twice and reactions were performed in duplicate.

These patients were classified according to severity and extension of the disease: fourteen patients had generalized PD and 16 had localized PD; regarding disease severity, 8 patients were classified as slight, 10 as moderate and 12 as severe PD. All patients and controls were nonsmokers, were not on anti-inflammatory drugs and had not taken antibiotics within the last 6 months and had no periodontal treatment during this same time.

According to the manufacturer’s instructions, the minimum detectable concentrations (lowest standard concentration) established for cytokines were: IL-1β, 9.81 pg/ml; IL-8, 12.21 pg/ml; TNF-α, 8.57 pg/ml; IL-6, 7.89 pg/ml; IL-2, 13.31 pg/ml; IL-5, 11.38 pg/ml; IFN-γ, 7.13 pg/ml; IL-4, 34.57 pg/ml; IL-10, 5.42 pg/ml; and GM-CSF, 6.86 pg/ml. When the values were below the sensitivity level, they were replaced by the lower detection threshold value for each analyte. In addition, when more than 50% of the samples had values below the sensitivity level, the analyte was excluded from further analysis and thus, some cytokines were not included in the results.

### Statistical analyses

Means and standard deviations were calculated using the OpenEpi software version 3.01 (http://www.openepi.com/Menu/OE_Menu.htm). The SNPStats software (https://www.snpstats.net/start.htm?) was used to evaluate if the estimated genotype distribution between observed and expected frequencies was found in the Hardy-Weinberg equilibrium (HWE), for descriptive analysis, chi-square test and logistic regression. For these analyzes, when all subjects were included, adjustments for smoking habits was made to eliminate this confounding factor. Adjustments for gender were made for all subjects and for nonsmoking groups.

The association tests were performed for codominant, dominant, recessive, overdominant and log-additive genetic inheritance models and the best inheritance model was chosen according to the lowest Akaike information criteria (AIC) [52]. Linkage disequilibrium (LD) among SNPs present in the same chromosome was measured by standardized disequilibrium (D′) and squared correlation (r^2^) coefficients using expectation-maximization algorithm. A strong LD was considered when D′ > 0.85 and r^2^ > 0.33 [53]. The permutation test was calculated using Haploview software. The Bonferroni adjustment for multiple testing was not applied because all variants analyzed have been associated to periodontitis in other populations.

QUANTO 1.2.4 software was useful to calculate the statistical power [54]. Considering the total number of individuals, the less frequent SNP (frequency equal to 0.05 for *TNF* -238), a population risk of 50.0% and a genetic effect of 3.0 there was a statistical power of 83.0% with confidence level of 5.0%. The statistical power for nonsmokers was also calculated, at a power of 82.0% and a genetic effect of 4.0. The statistical power for smokers was not considered satisfactory due to the number of participants in this study, therefore, this group was not considered in the analyses.

The concentration of the cytokines in the serum was estimated from xPONENT^®^ 3.1 software (Luminex Software, Inc.), expressed as pg/mL and adjusted for dilution factor. The normality was checked by the Shapiro Wilk test. The Mann-Whitney U test (https://www.socscistatistics.com/tests/mannwhitney/default2.aspx) was used to analyze the correlation between cytokine concentrations and genotypes, and to compare different groups (patients *vs*. controls, between patients according to extension of the disease, between patients with different degrees of severity, and extension or severity of disease *vs*. controls). The *P* value of less than 5.0% was considered statistically significant in all analyzes.

## Results

The clinical characteristics of the individuals are described in Table 1. The patients and controls were matched by gender and age (*P* ≥ 0.05). Women were 53% of patients and 62% of control group. The mean age was 47.7 ± 8.7 and 45.5 ± 8.8 years for patients and controls, respectively. Most of the individuals who participated in the study were nonsmokers (65.6% for patients and 84.1% for controls). Differences were found for smoking habits: there were more smokers in the PD group than in the control (34.4% *vs*. 15.9%; *P* < 0.0001, OR = 2.78, CI = 1.72–4.49), and smoking was a risk factor for PD in women (*P* = 0.0001; OR = 3.80; CI = 1.89–7.65) but not for PD in men (*P* = 0.08). Eighty-two patients were classified according to extension and severity of the disease: about half of them had the generalized or localized form and, regarding severity, 24.4% had slight, 41.5% moderate and 34.1% severe PD.

**Table 1:** Characteristics of patients with periodontitis and controls.

Characteristics	Periodontitis	Controls	*P* value	OR (95%CI)
	**Total**			
	N = 186	N = 208		
	n (%)	n (%)		
**Gender**				
Male	88 (47.0)	79 (38.0)		
Female	98 (53.0)	129 (62.0)	NS	
**Age**				
Mean ± SD (year)	47.7 ± 8.7	45.5 ± 8.8	NS	
**Smoking**				
Nonsmokers	122 (65.6)	175 (84.1)		Ref.
Smokers	64 (34.4)	33 (15.9)	< 0.0001	2.78 (1.72–4.49)
	**Female**			
Nonsmokers	67 (68.4)	115 (89.2)		Ref.
Smokers	31 (31.6)	14 (10.8)	0.0001	3.80 (1.89–7.65)
	**Male**			
Nonsmokers	55 (62.5)	60 (75.9)		Ref.
Smokers	33 (37.5)	19 (24.1)	NS	1.89 (0.97–3.71)
**Extension**				
Generalized	42 (52.2)			
Localized	40 (48.8)			
**Severity**				
Slight	20 (24.4)			
Moderate	34 (41.5)			
Severe	28 (34.1)			

OR: Odds Ratio, CI: Confidence Interval, N: total number, n: number of individuals, SD: standard deviation, Ref: reference, NS: not significant.

The genotype frequency distributions were consistent with the HWE (*P* ≥ 0.05), except for *IL4*–33 which was not included in the association analysis. The SNPs allele and genotype frequency distributions in patients and controls (nonsmokers, all subjects and smokers) were shown in supporting information (S1 Table). Allele frequency distributions were similar for all SNPs analyzed between PD patients and controls. The genotype frequencies of *IL1A*, *IL1R*, *IL1RN*, *IL4RA*, *INFG*, *TGFB1*, *TNF*, *IL2*, *IL4*, *IL6* and *IL10* polymorphisms were also similar in patients and control (*P* ≥ 0.05; nonsmokers and all subjects).

SNPs whose genotype and haplotype differed statistically between patients and controls (nonsmokers and all subjects) are shown in Table 2. *NLRP3*, rs4612666, were analyzed only in nonsmokers and the T/C genotype was more frequent in patients than in controls in codominant, dominant and overdominant models. Considering the AIC value (52), the overdominant model was considered to be the best inheritance model (56.0% vs. 37.3%, *P* = 0.0029, OR = 2.13, CI = 1.29–3.53). The *IL1B* -511 T/T genotype was more frequent in nonsmoking patients than in nonsmoking controls (22.8% *vs*. 12.3%, *P* = 0.028, OR = 2.10, CI = 1.08–4.11) and in all patients than in all controls (23.8% *vs*. 14.0%, *P* = 0.03, OR = 1.85, CI = 1.05–3.26, adjusted for smoking habit), both in a recessive inheritance model (T/T compared to C/C-C/T). The GT haplotype of *IL2* (+166, -330) was more frequent in patients than in controls (48.0% *vs*. 35.3%, *P* = 0.0091, OR = 1.68, CI = 1.16–2.44 for nonsmokers, and 45.7% *vs*. 35.7%, *P* = 0.02, OR = 1.49, CI = 1.09–2.04, for all subjects) compared to the other haplotype (GG + TT + TG). This haplotype association was maintained after permutation test with 10,000 permutations (permutation *P* = 0.029). A strong linkage disequilibrium (D´ = 0.96, r^2^ = 0.41) was verified between these *IL2* SNPs.

**Table 2:** *NLRP3* and *IL1B* genotypes and *IL2* Haplotype Frequency Distributions in patients with periodontitis and controls. Nonsmokers and All Subjects were Analyzed Separately*.

	Nonsmokers						All subjects					
Polymorphisms/ Inheritance model	Periodontitis		Controls		OR (95%CI)	*P* value	Periodontitis		Controls		OR (95%CI)^a^	*P* value
**Genotypes**	n	%	n	%			n	%	n	%		
***NLRP3* rs4612666 T>C**	N = 106		N = 150									
Overdominant												
T/T-C/C	48	44.0	94	62.7	Ref.							
T/C	61	56.0	56	37.3	2.13 (1.29–3.53)	0.0029						
***IL1B* -511C>T**	N = 111		N = 138				N = 172		N = 171			
Recessive												
C/C-C/T	88	77.2	121	87.7	Ref.		131	76.2	147	86.0	Ref.	
T/T	26	22.8	17	12.3	2.10 (1.08–4.11)	0.028	41	23.8	24	14.0	1.85 (1.05–3.26)	0.03
**Haplotype**^b^	*n*	%	*n*	%			*n*	%	*n*	%		
***IL2* (+166, −330)**^c^	*N* = 231		*N* = 231				*N* = 323		*N* = 323			
GG-TT-TG	120	52.0	149	64.7	Ref.		176	54.3	207	64.3	Ref.	
GT	111	48.0	82	35.3	1.68 (1.16–2.44)	0.0091^d^	147	45.7	116	35.7	1.49 (1.09–2.04)	0.02

OR: Odds Ratio, CI: Confidence Interval, N: total number, n: number of individuals, Ref.: the set of genotypes and haplotypes with higher frequency were used as reference group.

*Only significant results were shown.

^a^Adjusted for smoking habits.

^b^ *N*: total of haplotypes, *n*: number of haplotypes in each group.

^c^Linkage disequilibrium test of *IL2* +166 and -330: D´ = 0.96, r^2^ = 0.41.

^d^Nonsmokers: permutation *P* value = 0.029.

Considering gender and when only the men were analyzed, the *NLRP3* T/C genotype frequency was higher in nonsmoker PD compared with nonsmoker controls (*P* = 0.03, OR = 2.67, CI = 1.15–6.18; Table 3), and no differences in genotype frequency distributions were observed between women. No statistical differences were observed for other SNPs.

**Table 3:** Genotype and Haplotype Frequency Distributions for *NLRP3*, *IL1R*, *IL6*, *TNF*, *IL2*, *TGFB1*, *IL4* and *IL4RA* polymorphisms between periodontitis patients and controls, considering gender interaction*.

			Nonsmokers						All Subjects					
Gene/ Polymorphisms	Gender	Genotype	Periodontitis		Controls		OR (95%CI)	*P* value^a^	Periodontitis		Controls		OR (95%CI)^e^	*P* value^a^
**Genotypes**			n	%	n	%			n	%	n	%		
			N = 50		N = 58									
*NLRP3*	Male	C/C	15	30.0	29	50.0	Ref.							
rs4612666		T/C	29	58.0	21	36.2	2.67 (1.15–6.18)^b^	0.03						
		T/T	6	12.0	8	13.8								
			N = 51		N = 65									
*IL1R*	Female	C/T	25	49.0	45	69.2	Ref.							
+1970C>T	Male		26	51.0	20	30.8	2.34 (1.09–5.01)^c^	0.04						
			N = 40		N = 56				N = 60		N = 71			
*IL6*	Female	G/C	20	50.0	40	71.4	Ref.		27	45.0	47	66.2	Ref.	
-174G>C	Male		20	50.0	16	28.6	2.68 (1.13–6.33)^c^	0.05	33	55.0	24	33.8	2.14 (1.04–4.41)^c^	0.02
			N = 89		N = 106									
*TNF*	Female	G/G	49	55.1	75	70.8	Ref.							
-308G>A	Male		40	44.9	31	29.2	1.97 (1.09–3.57)^c^	0.03						
			N = 49		N = 62									
*IL2*	Female	T/T	24	49.0	45	72.6	Ref.							
-330T>G	Male		25	51.0	17	27.4	2.76 (1.25–6.08)^c^	0.01						
			N = 34		N = 42									
*TGFB1*	Female	T/T	18	52.9	34	81.0	Ref.							
+869T>C	Male		16	47.1	8	19.0	3.78 (1.36–10.51)^c^	0.02						
			N = 96		N = 117									
*TGFB1*	Female	G/G	52	54.2	84	71.8	Ref.							
+915G>C	Male		44	45.8	33	28.2	2.15 (1.22–3.80)^c^	0.01						
			N = 41		N = 57				N = 58		N = 72			
*IL4RA*	Female	G/A	18	43.9	44	77.2	Ref.		24	41,4	50	69.4	Ref.	
+1902G>A	Male		23	56.1	13	22.8	4.32 (1.81–10.36)^c^	0.001	34	58.6	22	30.6	2.85 (1.36–5.94)^c^	0.002
			N = 77		N = 97									
*IL4*	Female	T/T	43	55.8	71	73.2	Ref.							
-1098T>G	Male		34	44.2	26	26.8	2.16 (1.14–4.08)^c^	0.025						
			N = 49		N = 58									
*IL4*	Female	C/T	23	46.9	41	70.7	Ref.							
-590C>T	Male		26	53.1	17	29.3	2.73 (1.23–6.05)^c^	0.02						
**Haplotype**^f^			*n*	%	*n*	%			*n*	%	*n*	%		
			*N* = 146		*N* = 178				*N* = 232		*N* = 220			
*IL2* (+166, −330)	Female	GT	79	54.1	123	69.1	Ref.		119	51.3	142	64.5	Ref.	
	Male		67	45.9	55	30.9	3.89 (1.29–11.71)^d^	0.008	113	48.7	78	35.5	3.10 (1.22–7.88)^d^	0.006

OR: Odds Ratio, CI: Confidence Interval, Ref.: reference group, N: total number, n: number of individuals.

*Only significant results were shown.

^a^*P* value: Fisher exact test, univariate analysis.

^b^Between men.

^c^ Men were compared with women with the same genotype.

^d^Men were compared with women with the same haplotype.

^e^Adjusted for smoking habits.

^f^ *N*: total of haplotypes, *n*: number of haplotypes in each group.

After multivariate analysis (Table 3), when genotype frequency distribution in nonsmoking men was compared to the same genotype in nonsmoking women, differences (*P* < 0.05) were observed and the following genotypes were more frequent in men with PD: *IL1R* +1970 C/T (OR = 2.34, CI = 1.09–5.01), *IL6*–174 G/C (OR = 2.68, CI = 1.13–6.33), *TNF* -308 G/G (OR = 1.97, CI = 1.09–3.57), *IL2*–330 T/T (OR = 2.76, CI = 1.25–6.08), *TGFB1* +869 T/T (OR = 3.78, CI = 1.36–10.51), *TGFB* +915 G/G (OR = 2.15, CI = 1.22–3.80), *IL4RA* +1902 G/A (OR = 4.32, CI = 1.81–10.36), *IL4* −1098 T/T (OR = 2.16, CI = 1.14–4.08), *IL4*–590 C/T (OR = 2.73, CI = 1.23–6.05). When considering all subjects, the *IL6*–174 G/C (OR = 2.14, CI = 1.04–4.41) and *IL4RA* +1902 G/A (OR = 2.85, CI = 1.36–5.94) were also more frequent in men when compared to the same genotype in women. The GT haplotype of *IL2* (+166, -330) was also higher in men compared to women carrying the same haplotype (OR = 3.89, CI = 1.29–11.71 for nonsmokers; OR = 3.10, CI = 1.22–7.88 for all subjects).

Considering the sensitivity of the method used to detect serum cytokine concentrations, in addition to using half-diluted serum, not all cytokine serum levels could be detected in patients and controls. Thus, serum concentration was evaluated only for IL-8, IFN-γ, IL-4, IL-10 and GM-CSF (Table 4). Differences in the cytokine serum levels were not observed when patients were compared to controls and when genotypes were correlated with cytokine concentration. However, there was a tendency for the IL-4 serum levels to decrease in patients compared to controls (*P* = 0.057).

**Table 4:** Cytokine levels in serum of patients with periodontitis, classified according to extension and severity, and controls.

		Extension		Severity				
Cytokines	PD N = 30	Localized N = 16	Generalized N = 14	Slight N = 18	Moderate N = 10	Severe N = 12	Controls N = 8	*P* value*
IL-8**	15.45 (12.21–1437.50)	34.57 (5.58–1437.50)	15.45 (12.21–382.10)	237.73 (12.21–1437.50)	34.42 (12.21–230.16)	12.21 (5.58–66.99)^†^	69.43 (12.21–76.82)	0.60^a^
**IFN-γ**	18.57 (7.13–166.58)	18.57 (7.13–44.37)	18.57 (7.13–166.58)	22.99 (7.13–44.17)	22.99 (9.73–166.58)	14.15 (7.13–44.37)	22.99 (7.13–39.82)	**0.04**^b^
**IL-4**	51.24 (34.57–2455.95)	51.24 (34.57–2261.92)	51.24 (34.57–2455.95)	42.90 (34.57–140.12)	51.24 (34.57–2455.95)	51.24 (34.57–2261.92)	69.43 (51.24–244.85)	**0.02**^c^
**IL-10**	9.7 (5.42–191.55)	8.47 (5.42–191.55)	9.7 (5.42–47.42)	5.42 (5.42–191.55)^†^	7.56 (5.42–47.42)	9.7 (5.42–14.28)	9.7 (5.42–18.87)	0.62^a^
**GM-CSF**	6.80 (6.25–33.14)	6.80 (6.25–33.14)	6.80 (6.25–11.90)	6.80 (6.25–33.14)	7.07 (6.25–11.90)	6.80 (6.25–7.35)	6.80 (6.26–7.90)	0.59^a^

Cytokines levels detected in human serum. Data are expressed as median (range: minimum—maximum) and pg/mL. PD: periodontitis, N: number of individuals.

The analyses were done between: Patients *vs*. controls, localized *vs*. generalized, slight *vs*. moderate, slight *vs*. severe, moderate *vs*. severe, and extension or severity of disease *vs*. controls.

Limits of detection: IL-8, 12.21 pg/ml; IFN-γ, 7.13 pg/ml; IL-4, 34.57 pg/ml; IL-10, 5.42 pg/ml; and GM-CSF, 6.25 pg/ml.

^a^ Comparison between PD and control.

^b^ IFN-γ: Comparison between severity: moderate compared to severe degree of PD.

^c^ IL-4: Comparison between severity and controls: slight compared to control.

^†^ More than 50% of samples had values below the sensitivity level.

* Mann-Whitney U nonparametric test.

**IL-8: The median concentration of IL-8 was higher in the slight degree of the disease in relation to other groups, but no statistical differences were observed. The outlier was maintained and the wide range was considered in the analysis.

Differences were found when patients were grouped according to severity of PD (slight, moderate and severe). The concentration of IFN-γ was lower in the serum of patients with the severe degree of the disease when compared to the moderate degree (*P* = 0.04). The IL-4 serum levels were lower in patients with slight degree of PD when compared to the controls (*P* = 0.02).

## Discussion

In order to contribute to a better understanding of the complex mechanisms involved in immunopathogenesis of periodontitis, polymorphisms in inflammasome and cytokines genes were analyzed in this case-control study. Although the influence of *NLRP3*, *IL1B* and *IL2* polymorphisms in the development of PD have been reported in other populations [11,55], this is the first study involving these SNPs in PD patients and controls from southern of Brazil. A careful selection of the participants in this study and a judicious analysis of the data were considered.

We found that *NLRP3* (rs4612666) T/C genotype, *IL1B* -511 T/T genotype and *IL2* GT (+166, -330) haplotype were associated to susceptibility to PD, regardless of smoking habits. In addition, many immune gene variants were considered susceptibility factors for PD development in men but not in women: *NLRP3* T/C, *IL1R* +1970 C/T, *IL6*–174 G/C, *TNF* -308 G/G, *IL2*–330 T/T, *TGFB1* +869 T/T, *TGFB* +915 G/G, *IL4RA* +1902 G/A, *IL4*–1098 T/T and *IL4*–590 C/T genotypes, and *IL2* +166, -330 GT haplotype.

Some environmental and biological risk factors were previously associated with the development of PD, including smoking habits and gender [56]. Individuals who smoke cigarettes have a higher risk of developing PD, its severe form and have minor response to treatment compared to those who never smoked [57]. Consequences of smoking habits include immunosuppression and impaired cell functions such as in tissue repair promoted by fibroblasts [58,59]. In our study smoking cigarettes was a risk factor for the disease. In order to avoid bias, we analyzed only nonsmoking patients versus nonsmoking controls, and smoking was an adjustment variable when all individuals (smokers and nonsmokers) were analyzed. At this time, men have been considered to be more susceptible to periodontitis than women due to hormonal factors, personal hygiene habits and poor health prevention habits [60]. So, patients and controls were matched by gender.

In this study, *NLRP3* T/C genotype was associated with the risk of PD in nonsmokers. To our knowledge, only one study was conducted to investigate the influence of *NLRP3* polymorphism in periodontitis: it was in a Colombian cohort with similar results to ours, where the authors found that *NLRP3* T/C genotype was a risk factor for PD [55]. The *NLRP3* mutated allele (C) is a variant in the intron 7 of chromosome 1q44 and was previously correlated to the higher transcriptional activity of the gene when compared to the wild T allele [61]. An over expression of the NLRP3 inflammasome and a downregulation of NLRP3 inhibitors were observed in the gingival tissue of patients with PD [28,31,39]. The NLRP3 activation depends on signals provided by recognition of PAMPs, such as microbial lipopolysaccharides (LPS), and DAMPs (ie. extracellular adenosine triphosphate—ATP) through PRRs [62]. To confirm this biological mechanism, *in vitro* studies have shown that periodontopathogenic bacteria, such *Porphyromonas gingivalis*, *Aggregatibacter actinomycetemcomitans* and *Fusobacterium nucleatum*, are involved in the increased expression of NLRP3. The higher expression of NLRP3 stimulates the maturation and secretion of IL-1β [38,63,64] and IL-18 [32,63], and the pyroptic cell death [38] leading to an exacerbated inflammation in the periodontium tissue. However, the induction of NLRP3 also involves endogenous host factors such as ATP released by dying or injury of the cells [65]. Thus, in addition to the signals provide by the pathogen for NLRP3 activation, the polymorphism related to higher transcriptional activity of the *NLRP3* gene should be consider to better understand the inflammation pathway in the pathogenesis of PD.

IL-1β production is regulated by the inflammasome complex. IL-1β is a potent inflammatory mediator and bone-resorbing cytokine. This cytokine induces the chemotaxis of neutrophils and macrophages, the production of prostaglandin E2 and metalloproteinases, and the activation of lymphocytes and osteoclasts [66]. The higher IL-1β secretion was previously correlated to -511 T/T genotype [67]. We found that the *IL1B* -511 T/T genotype was associated to the risk of periodontitis in nonsmokers and in all subjects in this Southern Brazilian population. Previous findings had showed that the T allele was associated to periodontitis in Afro-Americans and Mulattos from the southeastern region of Brazil [68] and also in the Chinese population as shown in two meta-analysis studies [8,9]. The T/T genotype in codominant (T/T *vs*. C/C) and dominant (C/T + T/T *vs*. C/C) inheritance models were also associated to PD in Chinese [8]. Otherwise, in a cohort study conducted in Japanese pregnant women, the -511 C/T genotype showed a protective association to periodontitis after the adjustment of the odds ratio [69]. Other studies found no association between *IL1B* -511 polymorphism and periodontitis in southeastern Brazilian populations and in Indian [10,70]. As previously described, the IL-1β levels do not always decrease after periodontal therapy [24,25], thus, the knowledge that intrinsic host factors upregulate the inflammasome pathway and IL-1β production can be considered.

Other important finding in the study was that the haplotype composed by the *IL2* wild alleles (G at +166 and T at -330 positions) was a susceptibility factor for the development of periodontitis, although no association was observed for alleles and genotypes when each SNP was separately analyzed. Because these SNPs were in linkage disequilibrium in this studied population, the relevance of the biological function could occur when they were inherited together. In a study conducted in another Brazilian population, the *IL2–*330 T/T was associated to severity of PD in the dominant inheritance model (T/T *vs*. T/G+G/G) [11]. Differently, in Chinese, *IL2*–330 G allele and G/G genotype were a risk for periodontitis and were linked to higher levels of the IL-2 in the serum of these patients [71]. Another study showed that the *IL2* T/T+T/T genotypes (+166 and -330) when present in the same haplotype were factors of susceptibility to PD and were associated with higher burden of *P*. *gingivalis* and other bacteria from the red complex (*Tannerella forsythensis and Treponema denticola*) in the oral cavity; but when the *IL2–*330 T/T was individually analyzed, it was a protective factor for PD [72]. In Iranians, no association was found between *IL2*–330 and PD [73]. *In vitro*, T cells of individuals with *IL2*–330 G/G genotype was associated with higher IL-2 production when compared to cells of individuals with T/G and T/T genotypes [72]. However, there is no consensus about *IL2* +166 polymorphism and IL-2 production. IL-2 is a pro-inflammatory cytokine that mediates the activation, growth and differentiation of T cell subsets, B lymphocytes and NK cells directing the cellular immunity against periodontopathogenic bacteria [42]. *In vitro*, the decrease in IL-2 production was related to a reduction in the functional capacity of T lymphocytes from the periodontal tissue [74]. Moreover, periodontophatogenic bacteria such as *A*. *actinomycetemcomitans* and *F*. *nucleatum* may develop evasion mechanisms by inducing a suppression of cell-mediated immunity [75,76]. Thus, we suppose that the haplotype related to lower IL-2 production could induce a downregulation of specific immune response, giving an opportunity for bacterial plaque growth.

After multivariate analysis we observed a tendency (limited by the low statistical power) of men to be predisposed to the disease when in the presence of some polymorphisms in the inflammasome, cytokine and cytokine receptor genes, regardless of cigarette use. Previous studies had found a correlation between immune gene polymorphisms and men’s susceptibility to PD [77,78], including Southern Brazilians patients [13,79]. Thus, our data corroborates in emphasizing the importance of genetic factors in men’s susceptibility to developing PD.

As to cytokine concentration analysis, GM-CSF, IL-8, IFN-γ, IL-4 and IL-10 were measured in the serum and no differences were found in the cytokine levels between patients and controls. These results were consistent with others [80–83]. However, when patients were classified according to disease severity, the IFN-γ was lower in the serum of patients with severe PD compared to those with moderate PD (although not significant with slight severity and controls). Other studies have shown higher IFN-γ in serum of patients with PD compared to controls [84,85]. IFN-γ acts on neutrophils and macrophage activation controlling periodontal infection and decreasing *A*. *actinomycetemcomitans* infection in mice [86]. In addition, IFN-γ acts on Th1 cells signalization, promoting their differentiation and inducing proinflammatory cytokines production [87]. This cytokine may also inhibit osteoclastogenesis and control bone resorption, as previously shown [85,88,89]. Lower concentration of IFN-γ could favor an uncontrolled infection or/and leading to more tissue damage. Added to this, IL-4 levels were lower in patients with slight PD when compared to controls, and this was consistent with previous studies [90,91]. Lower IL-4 levels were also found when PD patients had smoking habits and/or diabetes [91]. IL-4 produced at sites of infection can induce Th2 lymphocyte differentiation and activate antibody production by B cells and inhibit the Th1 response [92]; the Th2 response decreased bone loss in PD [93].

This study intends to assist in some points that may be unclear regarding the immunopathogenesis of PD. First point, not all individuals who are in contact with oral pathogen develop the disease. Second, downregulation of the immune system molecules does not always occur after periodontal therapy. Third, does the greater predisposition of men to develop the periodontitis go beyond biological factors? It is known that genetic factors have an important influence in the innate and adaptive immune system and contribute to the different responses in individuals with PD. We demonstrated that the genotype of high IL-1β production and the genotype related to high transcriptional activity of *NLRP3* were associated with PD susceptibility. It is possibly that these genetic variations influence PD by upregulating the IL-1β production, a proinflammatory cytokine related to periodontal tissue damage. We also showed that *IL2* haplotype, related to low IL-2 production, was related to PD susceptibility. Downregulation of IL-2 could be corroborating in the suppression of cell-mediated immunity and causing susceptibility to infection. In addition, our study helped in understanding the relationship between man and periodontitis by demonstrating that polymorphisms in *NLRP3* and cytokine genes are associated with the risk of developing PD.

This study had limitations due to the low number of individuals when analyzing subgroups and the non-pairing of samples in all analyzed genes, due to the lack of some samples in the course of the study. In addition, it was not possible to determine the serum levels of some cytokines, especially the IL-1β. Moreover, the concentration of cytokines was also not evaluated in GCF and saliva. The expression of the inflammasome and cytokine genes was not available in gingival tissue.

## Conclusions

The polymorphisms in *NLRP3*, *IL1B* and *IL2* genes influence the development of periodontitis, independently of smoking habits. In addition, the polymorphisms in *NLRP3*, *IL1R*, *TNF*, *IL6*, *IL2*, *IL4* and *IL4RA* genes were associated with periodontitis in the males.

## Supporting information

S1 Table Allele and genotype frequency distributions of *NLRP3*, *IL1A*, *IL1R*, *IL1RN*, *IL4RA*, *INFG*, *TGFB1*, *TNF*, *IL2*, *IL4*, *IL6* and *IL10* gene polymorphisms in patients with periodontitis and controls (nonsmokers, all subjects and smokers). (DOCX)
