# Supplementary material for: Influence of inflammasome NLRP3, and IL1B and IL2 gene polymorphisms in periodontitis susceptibility
Source: PLoS One. 2020 Jan 24;15(1):e0227905. doi: 10.1371/journal.pone.0227905 (PMC6980600; doi:10.1371/journal.pone.0227905)
Supplement: S1 Table — (DOCX) [file pone.0227905.s001.docx]

**S1 Table.** **Allele and Genotype Frequency Distributions of *NLRP3, IL1A*, *IL1R*, *IL1RN*, *IL4RA*, *INFG*, *TGFB1*, *TNF*, *IL2*, *IL4*, *IL6* and *IL10* Gene Polymorphisms in Patients with Periodontitis and Controls (Nonsmokers, All Subjects and Smokers).**

|  |  | **Nonsmokers** | |  |  |  | **All Subjects** | |  |  |  | **Smokers** | |  |  |
| --- | --- | --- | --- | --- | --- | --- | --- | --- | --- | --- | --- | --- | --- | --- | --- |
| **Gene, SNP, RefSNP** |  | **Periodontitis** | **Controls** | **OR (95% CI)** | ***P* value** |  | **Periodontitis** | **Controls** | **OR (95% CI)†** | ***P* value** |  | **Periodontitis** | **Controls** | **OR (95% CI)** | ***P* value** |
|  |  | **n (%)** | **n (%)** |  |  |  | **n (%)** | **n (%)** |  |  |  | **n (%)** | **n (%)** |  |  |
| ***NLRP3*, rs4612666*** |  |  |  |  |  |  |  |  |  |  |  |  |  |  |  |
| Genotype | C/C | 38 (34.9) | 72 (48.0) | Ref. |  |  |  |  |  |  |  |  |  |  |  |
|  | C/T | 61 (56.0) | 56 (37.3) | 2.06 (1.21-3.52) | 0.011 |  |  |  |  |  |  |  |  |  |  |
|  | T/T | 10 (9.2) | 22 (14.7) | 0.86 (0.37-2.00) |  |  |  |  |  |  |  |  |  |  |  |
|  |  |  |  |  |  |  |  |  |  |  |  |  |  |  |  |
|  | C/C+T/T | 48 (44.0) | 94 (62.7) | Ref. |  |  |  |  |  |  |  |  |  |  |  |
|  | T/C | 61 (56.0) | 56 (37.3) | 2.13 (1.29-3.53) | 0.0029 |  |  |  |  |  |  |  |  |  |  |
|  |  |  |  |  |  |  |  |  |  |  |  |  |  |  |  |
| Allele | C | 137 (63.0) | 200 (67.0) | Ref. |  |  |  |  |  |  |  |  |  |  |  |
|  | T | 81 (37.0) | 100 (33.0) | 1.28 (0.82-1.70) | 0.4191 |  |  |  |  |  |  |  |  |  |  |
| HWE |  | 0.063 | 0.065 |  |  |  |  |  |  |  |  |  |  |  |  |
|  |  |  |  |  |  |  |  |  |  |  |  |  |  |  |  |
| ***IL1*, -889, rs1800587** |  |  |  |  |  |  |  |  |  |  |  |  |  |  |  |
| Genotype | C/C | 60 (54.0) | 76 (53.9) | Ref. |  |  | 96 (55.5) | 92 (52.9) | Ref. |  |  | 36 (58.1) | 16 (48.5) | Ref. |  |
|  | C/T | 45 (40.5) | 54 (38.3) | 1.06 (0.63-1.78) | 0.73 |  | 69 (39.9) | 69 (39.7) | 0.95 (0.61-1.49) | 0.61 |  | 24 (38.7) | 15 (45.5) | 0.71 (0.30-1.70) | 0.61 |
|  | T/T | 6 (5.4) | 11 (7.8) | 0.69 (0.24-1.98) |  |  | 8 (4.6) | 13 (7.5) | 0.63 (0.24-1.60) |  |  | 2 (3.2) | 2 (6.1) | 0.44 (0.06-3.44) |  |
|  |  |  |  |  |  |  |  |  |  |  |  |  |  |  |  |
| Allele | C | 165 (74.0) | 206 (73.0) | Ref. |  |  | 261 (75.0) | 253 (73.0) | Ref. |  |  | 96 (77.0) | 47 (71.0) | Ref. |  |
|  | T | 57 (26.0) | 76 (27.0) | 0.93 (0.63-1.40) | 0.8269 |  | 85 (25.0) | 95 (27.0) | 0.87 (0.62-1.22) | 0.46 |  | 28 (23.0) | 19 (29.0) | 0.72 (0.35-1.52) | 0.44 |
|  |  |  |  |  |  |  |  |  |  |  |  |  |  |  |  |
| HWE |  | 0.62 | 0.83 |  |  |  | 0.41 | 1 |  |  |  | 0.71 | 0.69 |  |  |
|  |  |  |  |  |  |  |  |  |  |  |  |  |  |  |  |
| ***IL1B,* -511, rs16944** |  |  |  |  |  |  |  |  |  |  |  |  |  |  |  |
| Genotype | C/C | 44 (38.6) | 51 (37.0) | Ref. |  |  | 65 (37.1) | 64 (37.4) | Ref. |  |  | 21 (34.4) | 13 (39.4) | Ref. |  |
|  | C/T | 44 (38.6) | 70 (50.7) | 0.73 (0.42-1.27) | 0.047 |  | 69 (39.4) | 83 (48.5) | 0.82 (0.51-1.33) | 0.088 |  | 25 (41.0) | 13 (39.4) | 1.19 (0.45-3.12) | 0.88 |
|  | T/T | 26 (22.8) | 17 (12.3) | 1.77 (0.85-3.69) |  |  | 41 (23.4) | 24 (14.0) | 1.61 (0.87-3.00) |  |  | 15 (24.6) | 7 (21.2) | 1.33 (0.43-4.12) |  |
|  |  |  |  |  |  |  |  |  |  |  |  |  |  |  |  |
|  | C/C+C/T | 88 (77.2) | 121 (87.7) | Ref. |  |  | 134 (76.6) | 147 (86.0) | Ref. |  |  |  |  |  |  |
|  | T/T | 26 (22.8) | 17 (12.3) | 2.10 (1.08-4.11) | 0.028 |  | 41 (23.4) | 24 (14.0) | 1.79 (1.02-3.15) | 0.04 |  |  |  |  |  |
|  |  |  |  |  |  |  |  |  |  |  |  |  |  |  |  |
| Allele | C | 132 (58.0) | 172 (62.0) | Ref. |  |  | 199 (57.0) | 211 (62.0) | Ref. |  |  | 67 (55.0) | 39 (59.0) | Ref. |  |
|  | T | 96 (42.0) | 104 (38.0) | 1.20 (0.84-1.72) | 0.3581 |  | 151 (43.0) | 131 (38.0) | 1.22 (0.90-1.66) | 0.22 |  | 55 (45.0) | 27 (41.0) | 1.18 (0.62-2.28) | 0.69 |
|  |  |  |  |  |  |  |  |  |  |  |  |  |  |  |  |
| HWE |  | 0.034 | 0.47 |  |  |  | 0.0092 | 0.87 |  |  |  | 0.2 | 0.29 |  |  |
|  |  |  |  |  |  |  |  |  |  |  |  |  |  |  |  |
| ***IL1B*, 3962, rs1143634** |  |  |  |  |  |  |  |  |  |  |  |  |  |  |  |
| Genotype | C/C | 72 (66.1) | 84 (61.8) | Ref. |  |  | 113 (66.1) | 105 (62.1) | Ref. |  |  | 41 (66.1) | 21 (63.6) |  |  |
|  | C/T | 31 (28.4) | 43 (31.6) | 0.84 (0.48-1.47) | 0.78 |  | 50 (29.2) | 54 (31.9) | 0.85 (0.53-1.37) | 0.77 |  | 19 (30.6) | 11 (33.3) | 0.88 (0.36-2.20) | 0.96 |
|  | T/T | 6 (5.5) | 9 (6.6) | 0.78 (0.26-2.29) |  |  | 8 (4.7) | 10 (5.9) | 0.82 (0.31-2.17) |  |  | 2 (3.2) | 1 (3.0) | 1.02 (0.09-11.96) |  |
|  |  |  |  |  |  |  |  |  |  |  |  |  |  |  |  |
| Allele | C | 175 (80.0) | 211 (78.0) | Ref. |  |  | 276 (81.0) | 264 (78.0) | Ref. |  |  | 101 (81.0) | 53 (80.0) | Ref. |  |
|  | T | 43 (20.0) | 61 (22.0) | 0.85 (0.55-1.32) | 0.538 |  | 66 (19.0) | 74 (22.0) | 0.85 (0.59-1.24) | 0.46 |  | 23 (19.0) | 13 (20.0) | 0.93 (0.41-2.17) | 0.99 |
|  |  |  |  |  |  |  |  |  |  |  |  |  |  |  |  |
| HWE |  | 0.36 | 0.32 |  |  |  | 0.46 | 0.37 |  |  |  | 1 | 1 |  |  |
|  |  |  |  |  |  |  |  |  |  |  |  |  |  |  |  |
| ***IL1R*,1970, rs2234650** |  |  |  |  |  |  |  |  |  |  |  |  |  |  |  |
| Genotype | C/C | 41 (36.0) | 57 (40.1) | Ref. |  |  | 66 (37.3) | 70 (40.0) | Ref. |  |  | 25 (39.7) | 13 (39.4) | Ref. |  |
|  | C/T | 51 (44.7) | 65 (45.8) | 1.09 (0.63-1.88) | 0.51 |  | 79 (44.6) | 77 (44.0) | 1.11 (0.70-1.78) | 0.82 |  | 28 (44.4) | 12 (36.4) | 1.21 (0.47-3.14) | 0.57 |
|  | T/T | 22 (19.3) | 20 (14.1) | 1.53 (0.74-3.16) |  |  | 32 (18.1) | 28 (16.0) | 1.20 (0.65-2.23) |  |  | 10 (15.9) | 8 (24.2) | 0.65 (0.21-2.04) |  |
|  |  |  |  |  |  |  |  |  |  |  |  |  |  |  |  |
| Allele | C | 133 (58.0) | 179 (63.0) |  |  |  | 211 (60.0) | 217 (62.0) | Ref. |  |  | 78 (62.0) | 38 (58.0) | Ref. |  |
|  | T | 95 (42.0) | 105 (37.0) | 1.21 (0.85-1.74) | 0.3217 |  | 143 (40.0) | 133 (38.0) | 1.11 (0.82-1.50) | 0.57 |  | 48 (38.0) | 28 (42.0) | 0.84 (0.44-1.61) | 0.67 |
|  |  |  |  |  |  |  |  |  |  |  |  |  |  |  |  |
| HWE |  | 0.44 | 0.86 |  |  |  | 0.35 | 0.42 |  |  |  | 0.6 | 0.16 |  |  |
|  |  |  |  |  |  |  |  |  |  |  |  |  |  |  |  |
| ***IL1RN*, 11100, rs315952** |  |  |  |  |  |  |  |  |  |  |  |  |  |  |  |
| Genotype | T/T | 58 (51.8) | 69 (48.6) | Ref. |  |  | 92 (52.9) | 87 (49.7) | Ref. |  |  | 34 (54.8) | 18 (54.5) | Ref. |  |
|  | T/C | 44 (39.3) | 65 (45.8) | 0.81 (0.48-1.35) | 0.43 |  | 64 (36.8) | 77 (44.0) | 0.82 (0.52-1.29) | 0.36 |  | 20 (32.3) | 12 (36.4) | 0.88 (0.35-2.20) | 0.82 |
|  | C/C | 10 (8.9) | 8 (5.6) | 1.49 (0.55-4.01) |  |  | 18 (10.3) | 11 (6.3) | 1.46 (0.64-3.32) |  |  | 8 (12.9) | 3 (9.1) | 1.41 (0.33-5.99) |  |
|  |  |  |  |  |  |  |  |  |  |  |  |  |  |  |  |
| Allele | T | 160 (71.0) | 203 (71.0) | Ref. |  |  | 248 (71.0) | 251 (72.0) | Ref. |  |  | 88 (71.0) | 48 (73.0) | Ref. |  |
|  | C | 64 (29.0) | 81 (29.0) | 1 (0.68-1.48) | 1 |  | 100 (29.0) | 99 (28.0) | 1.02 (0.74-1.42) | 0.96 |  | 36 (29.0) | 18 (27.0) | 1.09 (0.53-2.27) | 0.94 |
|  |  |  |  |  |  |  |  |  |  |  |  |  |  |  |  |
| HWE |  | 0.65 | 0.22 |  |  |  | 0.35 | 0.19 |  |  |  | 0.12 | 0.67 |  |  |
|  |  |  |  |  |  |  |  |  |  |  |  |  |  |  |  |
| ***IL4RA*, 1902, rs1801275** |  |  |  |  |  |  |  |  |  |  |  |  |  |  |  |
| Genotype | A/A | 66 (58.9) | 72 (51.4) | Ref. |  |  | 107 (61.1) | 88 (50.9) | Ref. |  |  | 41 (65.1) | 16 (48.5) | Ref. |  |
|  | A/G | 41 (36.6) | 57 (40.7) | 0.78 (0.47-1.32) | 0.35 |  | 58 (33.1) | 72 (41.6) | 0.68 (0.43-1.07) | 0.19 |  | 17 (27.0) | 15 (45.5) | 0.44 (0.18-1.09) | 0.2 |
|  | G/G | 5 (4.5) | 11 (7.9) | 0.50 (0.16-1.50) |  |  | 10 (5.7) | 13 (7.5) | 0.62 (0.25-1.50) |  |  | 5 (7.9) | 2 (6.1) | 0.98 (0.17-5.55) |  |
|  |  |  |  |  |  |  |  |  |  |  |  |  |  |  |  |
| Allele | A | 173 (77.0) | 201 (72.0) | Ref. |  |  | 272 (78.0) | 248 (72.0) | Ref. |  |  | 99 (79.0) | 47 (71.0) | Ref. |  |
|  | G | 51 (23.0) | 79 (28.0) | 0.75 (0.49-1.13) | 0.198 |  | 78 (22.0) | 98 (28.0) | 0.72 (0.51-1.04) | 0.08 |  | 27 (21.0) | 19 (29.0) | 0.67 (0.32-1.42) | 0.34 |
|  |  |  |  |  |  |  |  |  |  |  |  |  |  |  |  |
| HWE |  | 0.79 | 1 |  |  |  | 0.52 | 0.85 |  |  |  | 0.13 | 0.69 |  |  |
|  |  |  |  |  |  |  |  |  |  |  |  |  |  |  |  |
| ***IFNG*, 874, rs2430561** |  |  |  |  |  |  |  |  |  |  |  |  |  |  |  |
| Genotype | A/A | 37 (33.9) | 46 (33.1) | Ref. |  |  | 58 (34.3) | 56 (32.6) | Ref. |  |  | 21 (35.0) | 10 (30.3) | Ref. |  |
|  | A/T | 59 (54.1) | 75 (54.0) | 0.98 (0.56-1.70) | 0.97 |  | 91 (53.9) | 88 (51.2) | 1.02 (0.63-1.64) | 0.34 |  | 32 (53.3) | 13 (39.4) | 1.17 (0.43-3.16) | 0.088 |
|  | T/T | 13 (11.9) | 18 (12.9) | 0.90 (0.39-2.07) |  |  | 20 (11.8) | 28 (16.3) | 0.63 (0.31-1.27) |  |  | 7 (11.7) | 10 (30.3) | 0.33 (0.10-1.13) |  |
|  |  |  |  |  |  |  |  |  |  |  |  |  |  |  |  |
| Allele | A | 133 (61.0) | 167 (60.0) | Ref. |  |  | 207 (61.0) | 200 (58.0) | Ref. |  |  | 74 (62.0) | 33 (0.5) | Ref. |  |
|  | T | 85 (39.0) | 111 (40.0) | 0.96 (0.67-1.38) | 0.905 |  | 131 (39.0) | 144 (42.0) | 0.88 (0.64-1.21) | 0.45 |  | 46 (38.0) | 33 (0.5) | 0.62 (0.32-1.19) | 0.17 |
|  |  |  |  |  |  |  |  |  |  |  |  |  |  |  |  |
| HWE |  | 0.23 | 0.16 |  |  |  | 0.11 | 0.54 |  |  |  | 0.42 | 0.29 |  |  |
|  |  |  |  |  |  |  |  |  |  |  |  |  |  |  |  |
| ***TGFB*, 869, rs1982073** |  |  |  |  |  |  |  |  |  |  |  |  |  |  |  |
| Genotype | C/C | 20 (18.4) | 25 (17.9) | 0.99 (0.47-2.07) |  |  | 87 (50.9) | 91 (52.6) | 1.06 (0.56-2.02) |  |  | 12 (19.4) | 5 (15.2) | 1.33 (0.36-4.88) |  |
|  | C/T | 55 (50.5) | 73 (52.1) | 0.93 (0.53-1.65) | 0.97 |  | 32 (18.7) | 30 (17.3) | 0.94 (0.58-1.55) | 0.92 |  | 32 (51.6) | 18 (54.5) | 0.99 (0.38-2.59) | 0.88 |
|  | T/T | 34 (31.2) | 42 (30.0) | Ref. |  |  | 52 (30.4) | 52 (30.1) | Ref |  |  | 18 (2.09) | 10 (30.3) | Ref. |  |
|  |  |  |  |  |  |  |  |  |  |  |  |  |  |  |  |
| Allele | C | 95 (44.0) | 123 (44.0) | 0.98 (0.69-1.41) | 1 |  | 151 (44.0) | 151 (44.0) | 0.97 (0.72-1.34) | 0.95 |  | 56 (45.0) | 28 (42.0) | 1.12 (0.58-2.14) | 0.83 |
|  | T | 123 (56.0) | 157 (56.0) | Ref. |  |  | 191 (56.0) | 195 (56.0) | Ref. |  |  | 68 (55.0) | 38 (58.0) | Ref. |  |
|  |  |  |  |  |  |  |  |  |  |  |  |  |  |  |  |
| HWE |  | 0.85 | 0.61 |  |  |  | 0.76 | 0.44 |  |  |  | 0.8 | 0.72 |  |  |
|  |  |  |  |  |  |  |  |  |  |  |  |  |  |  |  |
| ***TGFB*, 915, rs1800471** |  |  |  |  |  |  |  |  |  |  |  |  |  |  |  |
| Genotype | G/G | 96 (88.1) | 117 (84.2) | Ref. |  |  | 149 (87.1) | 147 (85.5) | Ref. |  |  | 53 (85.5) | 30 (90.9) | Ref. |  |
|  | G/C | 13 (11.9) | 20 (14.4) | 0.79 (0.37-1.67) | 0.26 |  | 22 (12.9) | 23 (13.4) | 0.95 (0.50-1.80) | 0.31 |  | 9 (14.5) | 3 (9.1) | 1.70 (0.43-6.76) | 0.44 |
|  | C/C | 0 (0.0) | 2 (1.4) | 0.40 (0.04-3.96) |  |  | 0 (0.0) | 2 (1.2) |  |  |  | 0 (0.0) | 0 (0.0) |  |  |
|  |  |  |  |  |  |  |  |  |  |  |  |  |  |  |  |
| Allele | G | 205 (94.0) | 254 (91.0) | Ref. |  |  | 320 (94.0) | 317 (92.0) | Ref. |  |  | 115 (93.0) | 63 (95.0) | Ref. |  |
|  | C | 13 (0.6) | 24 (0.9) | 0.67 (0.33-1.35) | 0.342 |  | 22 (6.0) | 27 (8.0) | 0.83 (0.44-1.55) | 0.64 |  | 9 (7.0) | 3 (5.0) | 1.64 (0.39-9.75) | 0.69 |
|  |  |  |  |  |  |  |  |  |  |  |  |  |  |  |  |
| HWE |  | 1 | 0.26 |  |  |  | 1 | 0.27 |  |  |  | 1 | 1 |  |  |
|  |  |  |  |  |  |  |  |  |  |  |  |  |  |  |  |
| ***TNF*, -308, rs1800629** |  |  |  |  |  |  |  |  |  |  |  |  |  |  |  |
| Genotype | G/G | 89 (80.2) | 106 (74.7) | Ref. |  |  | 137 (80.6) | 132 (75.4) | Ref. |  |  | 48 (81.4) | 26 (78.8) | Ref. |  |
|  | G/A | 20 (18.0) | 32 (22.5) | 0.74 (0.40-1.39) | 0.56 |  | 30 (17.6) | 38 (21.7) | 0.78 (0.45-1.34) | 0.53 |  | 10 (16.9) | 6 (18.2) | 0.90 (0.29-2.76) | 0.9 |
|  | A/A | 2 (1.8) | 4 (2.8) | 0.60 (0.11-3.33) |  |  | 3 (1.8) | 5 (2.9) | 0.58 (0.13-2.53) |  |  | 1 (1.7) | 1 (3.0) | 0.54 (0.03-9.02) |  |
|  |  |  |  |  |  |  |  |  |  |  |  |  |  |  |  |
| Allele | G | 198 (89.0) | 244 (86.0) | Ref. |  |  | 304 (89.0) | 302 (86.0) | Ref. |  |  | 106 (90.0) | 58 (88.0) | Ref. |  |
|  | A | 24 (11.0) | 40 (14.0) | 0.74 (0.43-1.27) | 0.334 |  | 36 (11.0) | 48 (14.0) | 0.74 (0.45-1.21) | 0.25 |  | 12 (10.0) | 8 (12.0) | 0.82 (0.29-2.46) | 0.86 |
|  |  |  |  |  |  |  |  |  |  |  |  |  |  |  |  |
| HWE |  | 0.61 | 0.48 |  |  |  | 0.40 | 0.33 |  |  |  | 0.46 | 0.38 |  |  |
|  |  |  |  |  |  |  |  |  |  |  |  |  |  |  |  |
| ***TNF*, -238, rs361525** |  |  |  |  |  |  |  |  |  |  |  |  |  |  |  |
| Genotype | G/G | 97 (87.4) | 123 (86.6) | Ref. |  |  | 152 (89.4) | 153 (87.4) | Ref. |  |  | 55 (93.2) | 30 (90.9) | Ref. |  |
|  | G/A | 13 (11.7) | 19 (13.4) | 0.87 (0.41-1.84) | 0.41 |  | 16 (9.4) | 22 (12.6) | 0.80 (0.40-1.60) | 0.23 |  | 3 (5.1) | 3 (9.1) | 0.55 (0.10-2.87) | 0.5 |
|  | A/A | 1 (0.9) | 0 (0.0) | 2.53 (0.23-28.3) |  |  | 2 (1.2) | 0 (0.0) |  |  |  | 1 (1.7) | 0 (0.0) | 1.11 (0.05-67.4) |  |
|  |  |  |  |  |  |  |  |  |  |  |  |  |  |  |  |
| Allele | G | 207 (93.0) | 265 (93.0) | Ref. |  |  | 320 (94.0) | 328 (94.0) | Ref. |  |  | 113 (96.0) | 63 (95.0) | Ref. |  |
|  | A | 15 (0.7) | 19 (0.7) | 1 (0.50-2.04) | 1 |  | 20 (6.0) | 22 (6.0) | 0.93 (0.50-1.74) | 0.95 |  | 5 (4.0) | 3 (5.0) | 0.93 (0.17-6.18) | 0.99 |
|  |  |  |  |  |  |  |  |  |  |  |  |  |  |  |  |
| HWE |  | 0.4 | 1 |  |  |  | 0.1 | 1 |  |  |  | 0.084 | 1 |  |  |
|  |  |  |  |  |  |  |  |  |  |  |  |  |  |  |  |
| ***IL2*, -330, rs2069762** |  |  |  |  |  |  |  |  |  |  |  |  |  |  |  |
| Genotype | T/T | 49 (50.0) | 62 (46.6) | Ref. |  |  | 79 (50.0) | 77 (46.7) | Ref. |  |  | 30 (50.0) | 15 (46.9) | Ref. |  |
|  | T/G | 44 (44.9) | 58 (43.6) | 0.96 (0.56-1.65) | 0.40 |  | 65 (41.1) | 74 (44.9) | 0.87 (0.54-1.39) | 0.84 |  | 21 (35.0) | 16 (50.0) | 0.66 (0.27-1.61) | 0.11 |
|  | G/G | 5 (5.1) | 13 (9.8) | 0.49 (0.16-1.46) |  |  | 14 (8.9) | 14 (8.5) | 0.91 (0.40-2.08) |  |  | 9 (15.0) | 1 (3.1) | 4.50 (0.52-38.90) |  |
|  |  |  |  |  |  |  |  |  |  |  |  |  |  |  |  |
| Allele | T | 142 (72.0) | 182 (68.0) | Ref. |  |  | 223 (71.0) | 228 (69.0) |  |  |  | 81 (68.0) | 46 (72.0) | Ref. |  |
|  | G | 54 (28.0) | 84 (32.0) | 0.82 (0.55-1.24) | 0.406 |  | 93 (29.0) | 102 (31.0) | 0.93 (0.66-1.32) | 0.75 |  | 39 (32.0) | 18 (28.0) | 1.23 (0.60-2.56) | 0.66 |
|  |  |  |  |  |  |  |  |  |  |  |  |  |  |  |  |
| HWE |  | 0.31 | 1 |  |  |  | 0.85 | 0.59 |  |  |  | 0.14 | 0.38 |  |  |
|  |  |  |  |  |  |  |  |  |  |  |  |  |  |  |  |
| ***IL2*, 166, rs2069763** |  |  |  |  |  |  |  |  |  |  |  |  |  |  |  |
| Genotype | G/G | 56 (57.1) | 63 (47.4) | Ref. |  |  | 88 (55.7) | 78 (47.3) | Ref. |  |  | 32 (53.3) | 15 (46.9) | Ref. |  |
|  | G/T | 34 (34.7) | 52 (39.1) | 0.74 (0.42-1.29) | 0.24 |  | 56 (35.4) | 64 (38.8) | 0.77 (0.47-1.24) | 0.18 |  | 22 (36.7) | 12 (37.5) | 0.86 (0.34-2.19) | 0.70 |
|  | T/T | 8 (8.2) | 18 (13.5) | 0.50 (0.20-1.24) |  |  | 14 (8.9) | 23 (13.9) | 0.52 (0.24-1.09) |  |  | 6 (10.0) | 5 (15.6) | 0.56 (0.15-2.14) |  |
|  |  |  |  |  |  |  |  |  |  |  |  |  |  |  |  |
| Allele | G | 146 (74.0) | 178 (67.0) | Ref. |  |  | 232 (73.0) | 220 (67.0) | Ref. |  |  | 86 (72.0) | 42 (66.0) | Ref. |  |
|  | T | 50 (26.0) | 88 (33.0) | 0.69 (0.46-1.04) | 0.097 |  | 84 (27.0) | 110 (33.0) | 0.72 (0.51-1.03) | 0.07 |  | 34 (28.0) | 22 (34.0) | 0.75 (0.37-1.53) | 0.49 |
|  |  |  |  |  |  |  |  |  |  |  |  |  |  |  |  |
| HWE |  | 0.42 | 0.17 |  |  |  | 0.31 | 0.11 |  |  |  | 0.52 | 0.43 |  |  |
|  |  |  |  |  |  |  |  |  |  |  |  |  |  |  |  |
| ***IL4*, -1098, rs22432484** |  |  |  |  |  |  |  |  |  |  |  |  |  |  |  |
| Genotype | T/T | 77 (77.0) | 97 (75.2) | Ref. |  |  | 115 (73.2) | 121 (75.2) | Ref. |  |  | 38 (66.7) | 24 (75.0) | Ref. |  |
|  | T/G | 23 (23.0) | 32 (24.8) | 0.91 (0.49-1.67) | 0.75 |  | 40 (25.5) | 40 (24.8) | 1.01 (0.60-1.70) | 0.4 |  | 17 (29.8) | 8 (25.0) | 1.34 (0.50-3.59) | 0.34 |
|  | G/G | 0 (0.0) | 0 (0.0) |  |  |  | 2 (1.3) | 0 (0.0) |  |  |  | 2 (3.5) | 0 (0.0) | 1.91 (0.14-104.9) |  |
|  |  |  |  |  |  |  |  |  |  |  |  |  |  |  |  |
| Allele | T | 177 (88.0) | 226 (88.0) | Ref. |  |  | 270 (86.0) | 282 (88.0) |  |  |  | 93 (82.0) | 56 (88.0) | Ref. |  |
|  | G | 23 (12.0) | 32 (12.0) | 0.91 (0.52-1.62) | 0.8845 |  | 44 (14.0) | 40 (12.0) | 1.15 (0.72-0.82) | 0.63 |  | 21 (18.0) | 8 (12.0) | 1.58 (0.62-4.40) | 0.42 |
|  |  |  |  |  |  |  |  |  |  |  |  |  |  |  |  |
| HWE |  | 0.35 | 0.22 |  |  |  | 0.74 | 0.14 |  |  |  | 1 | 1 |  |  |
|  |  |  |  |  |  |  |  |  |  |  |  |  |  |  |  |
| ***IL4*, -590, rs2243250** |  |  |  |  |  |  |  |  |  |  |  |  |  |  |  |
| Genotype | C/C | 40 (40.4) | 61 (47.3) | Ref. |  |  | 66 (42.3) | 81 (50.3) | Ref. |  |  | 26 (45.6) | 20 (62.5) | Ref. |  |
|  | C/T | 49 (49.5) | 58 (45.0) | 1.29 (0.74-2.24) | 0.55 |  | 74 (47.4) | 70 (43.5) | 1.38 (0.86-2.21) | 0.14 |  | 25 (43.9) | 12 (37.5) | 1.60 (0.65-3.95) | 0.05 |
|  | T/T | 10 (10.1) | 10 (7.8) | 1.52 (0.58-3.99) |  |  | 16 (10.3) | 10 (6.2) | 2.16 (0.91-5.16) |  |  | 6 (10.5) | 0 (0.0) | 5.31 (0.62-47.75) |  |
| Allele |  |  |  |  |  |  |  |  |  |  |  |  |  |  |  |
|  | C | 129 (65.0) | 180 (70.0) | Ref. |  |  | 206 (66.0) | 232 (72.0) | Ref. |  |  | 77 (68.0) | 52 (81.0) | Ref. |  |
|  | T | 69 (35.0) | 78 (30.0) | 1.23 (0.83-1.83) | 0.345 |  | 106 (34.0) | 90 (28.0) | 0.75 (0.53-1.07) | 0.12 |  | 37 (32.0) | 12 (19.0) | 2.07 (0.95-4.79) | 0.07 |
|  |  |  |  |  |  |  |  |  |  |  |  |  |  |  |  |
| HWE |  | 0.51 | 0.54 |  |  |  | 0.59 | 0.43 |  |  |  | 1 | 0.56 |  |  |
|  |  |  |  |  |  |  |  |  |  |  |  |  |  |  |  |
| ***IL4*, -33, rs2070874** |  |  |  |  |  |  |  |  |  |  |  |  |  |  |  |
| Genotype | C/C | 55 (56.1) | 71 (55.9) | Ref. |  |  | 82 (53.2) | 92 (57.9) | Ref. |  |  | 27 (48.2) | 21 (65.6) | Ref. |  |
|  | C/T | 29 (29.6) | 38 (29.9) | 0.99 (0.54-1.79) | 1 |  | 47 (30.5) | 48 (30.2) | 1.08 (0.65-1.80) | 0.5 |  | 18 (32.1) | 10 (31.2) | 1.40 (0.54-3.66) | 0.05 |
|  | T/T | 14 (14.3) | 18 (14.2) | 1.00 (0.46-2.20) |  |  | 25 (16.2) | 19 (11.9) | 1.50 (0.76-2.95) |  |  | 11 (19.6) | 1 (3.1) | 8.56 (1.02-71.58) |  |
|  |  |  |  |  |  |  |  |  |  |  |  |  |  |  |  |
| Allele | C | 139 (71.0) | 180 (71.0) | Ref. |  |  | 211 (69.0) | 232 (73.0) | Ref. |  |  | 72 (64.0) | 52 (81.0) | Ref. |  |
|  | T | 57 (29.0) | 74 (29.0) | 1.00 (0.66-1.50) | 1 |  | 97 (31.0) | 86 (27.0) | 1.24 (0.86-1.78) | 0.26 |  | 40 (36.0) | 12 (19.0) | 2.39 (1.10-5.52) | 0.02 |
|  |  |  |  |  |  |  |  |  |  |  |  |  |  |  |  |
| HWE |  | 0.0066 | 0.0024 |  |  |  | 0.00035 | 0.0044 |  |  |  | 0.039 | 1 |  |  |
|  |  |  |  |  |  |  |  |  |  |  |  |  |  |  |  |
| ***IL6*, -174, rs1800795** |  |  |  |  |  |  |  |  |  |  |  |  |  |  |  |
| Genotype | G/G | 56 (52.3) | 73 (52.5) | Ref. |  |  | 88 (53.0) | 87 (51.2) | Ref. |  |  | 32 (54.2) | 14 (45.2) | Ref. |  |
|  | G/C | 41 (38.3) | 56 (40.3) | 0.95 (0.56-1.63) |  |  | 60 (36.1) | 71 (41.8) | 0.83 (0.52-1.32) | 0.41 |  | 19 (32.2) | 15 (48.4) | 0.55 (0.22-1.40) | 0.26 |
|  | C/C | 10 (9.3) | 10 (7.2) | 1.30 (0.51-3.35) | 0.82 |  | 18 (10.8) | 12 (7.1) | 1.41 (0.63-3.16) |  |  | 8 (13.6) | 2 (6.5) | 1.75 (0.33-9.31) |  |
|  |  |  |  |  |  |  |  |  |  |  |  |  |  |  |  |
| Allele | G | 153 (71.0) | 202 (73.0) | Ref. |  |  | 236 (71.0) | 245 (72.0) | Ref. |  |  | 83 (70.0) | 43 (69.0) | Ref. |  |
|  | C | 61 (29.0) | 76 (27.0) | 1.06 (0.71-1.58) | 0.852 |  | 96 (29.0) | 95 (28.0) | 1.05 (0.74-1.49) | 0.84 |  | 35 (30.0) | 19 (31.0) | 0.95 (0.47-1.99) | 0.99 |
|  |  |  |  |  |  |  |  |  |  |  |  |  |  |  |  |
| HWE |  | 0.63 | 1 |  |  |  | 0.13 | 0.71 |  |  |  | 0.11 | 0.68 |  |  |
|  |  |  |  |  |  |  |  |  |  |  |  |  |  |  |  |
| ***IL6*, nt565, rs1800797** |  |  |  |  |  |  |  |  |  |  |  |  |  |  |  |
| Genotype | G/G | 59 (55.1) | 77 (55.8) | Ref. |  |  | 92 (55.4) | 91 (53.9) | Ref. |  |  | 33 (55.9) | 14 (45.2) | Ref. |  |
|  | G/A | 40 (37.4) | 54 (39.1) | 0.97 (0.57-1.64) | 0.73 |  | 59 (35.5) | 70 (41.4) | 0.82 (0.52-1.29) | 0.25 |  | 19 (32.2) | 16 (51.6) | 0.50 (0.20-1.26) | 0.11 |
|  | A/A | 8 (7.5) | 7 (5.1) | 1.49 (0.51-4.35) |  |  | 15 (9.0) | 8 (4.7) | 1.75 (0.70-4.41) |  |  | 7 (11.9) | 1 (3.2) | 2.97 (0.33-26.44) |  |
|  |  |  |  |  |  |  |  |  |  |  |  |  |  |  |  |
| Allele | G | 158 (74.0) | 208 (75.0) | Ref. |  |  | 243 (73.0) | 252 (75.0) | Ref. |  |  | 85 (72.0) | 44 (71.0) | Ref. |  |
|  | A | 56 (26.0) | 68 (25.0) | 1.08 (0.72-1.63) | 0.777 |  | 89 (27.0) | 86 (25.0) | 1.07 (0.75-1.54) | 0.75 |  | 33 (28.0) | 18 (29.0) | 0.95 (0.46-2.00) | 0.99 |
|  |  |  |  |  |  |  |  |  |  |  |  |  |  |  |  |
| HWE |  | 0.8 | 0.65 |  |  |  | 0.24 | 0.31 |  |  |  | 0.12 | 0.38 |  |  |
|  |  |  |  |  |  |  |  |  |  |  |  |  |  |  |  |
| ***IL10*, -1082, rs1800896** |  |  |  |  |  |  |  |  |  |  |  |  |  |  |  |
| Genotype | A/A | 51 (48.1) | 52 (37.7) | Ref. |  |  | 76 (46.1) | 67 (39.2) | Ref. |  |  | 25 (42.4) | 15 (45.5) | Ref. |  |
|  | A/G | 46 (43.4) | 72 (52.2) | 0.65 (0.38-1.11) | 0.26 |  | 77 (46.7) | 86 (50.3) | 0.79 (0.50-1.24) | 0.37 |  | 31 (52.5) | 14 (42.4) | 1.33 (0.54-3.26) | 0.41 |
|  | G/G | 9 (8.5) | 14 (10.1) | 0.66 (0.26-1.65) |  |  | 12 (7.3) | 18 (10.5) | 0.60 (0.27-1.36) |  |  | 3 (5.1) | 4 (12.1) | 0.45 (0.09-2.29) |  |
|  |  |  |  |  |  |  |  |  |  |  |  |  |  |  |  |
| Allele | A | 148 (70.0) | 176 (64.0) | Ref. |  |  | 229 (69.0) | 220 (64.0) | Ref. |  |  | 81 (69.0) | 44 (67.0) | Ref. |  |
|  | G | 64 (30.0) | 100 (36.0) | 0.76 (0.52-1.11) | 0.1917 |  | 101 (31.0) | 122 (36.0) | 0.79 (0.57-1.11) | 0.19 |  | 37 (31.0) | 22 (33.0) | 0.91 (0.46-1.84) | 0.91 |
|  |  |  |  |  |  |  |  |  |  |  |  |  |  |  |  |
| HWE |  | 1 | 0.2 |  |  |  | 0.27 | 0.25 |  |  |  | 0.13 | 0.71 |  |  |
|  |  |  |  |  |  |  |  |  |  |  |  |  |  |  |  |
| ***IL10*, -819, rs1800871** |  |  |  |  |  |  |  |  |  |  |  |  |  |  |  |
| Genotype | C/C | 56 (52.8) | 61 (44.2) | Ref. |  |  | 83 (50.3) | 78 (45.6) | Ref. |  |  | 27 (45.8) | 17 (51.5) | Ref. |  |
|  | C/T | 35 (33.0) | 59 (42.8) | 0.65 (0.37-1.12) | 0.29 |  | 60 (36.4) | 71 (41.5) | 0.78 (0.49-1.25) | 0.58 |  | 25 (42.4) | 12 (36.4) | 1.31 (0.52-3.28) | 0.84 |
|  | T/T | 15 (14.2) | 18 (13.0) | 0.91 (0.42-1.97) |  |  | 22 (13.3) | 22 (12.9) | 0.96 (0.49-1.88) |  |  | 7 (11.9) | 4 (12.1) | 1.10 (0.28-4.34) |  |
|  |  |  |  |  |  |  |  |  |  |  |  |  |  |  |  |
| Allele | C | 147 (69.0) | 181 (66.0) | Ref. |  |  | 226 (68.0) | 227 (66.0) | Ref. |  |  | 79 (67.0) | 46 (70.0) | Ref. |  |
|  | T | 65 (31.0) | 95 (34.0) | 0.84 (0.57-1.24) | 0.436 |  | 104 (32.0) | 115 (34.0) | 0.91 (0.65-1.27) | 0.62 |  | 39 (33.0) | 20 (30.0) | 1.13 (0.57-2.31) | 0.83 |
|  |  |  |  |  |  |  |  |  |  |  |  |  |  |  |  |
| HWE |  | 0.023 | 0.57 |  |  |  | 0.05 | 0.39 |  |  |  | 0.77 | 0.42 |  |  |
|  |  |  |  |  |  |  |  |  |  |  |  |  |  |  |  |
| ***IL10*, -592, rs1800872** |  |  |  |  |  |  |  |  |  |  |  |  |  |  |  |
| Genotype | C/C | 51 (47.7) | 59 (43.1) | Ref. |  |  | 75 (45.5) | 76 (44.7) | Ref. |  |  | 24 (41.4) | 17 (51.5) | Ref. |  |
|  | C/A | 39 (36.5) | 59 (43.1) | 0.76 (0.44-1.33) | 0.58 |  | 64 (38.8) | 70 (41.2) | 0.93 (0.58-1.49) | 0.88 |  | 25 (43.1) | 11 (33.3) | 1.61 (0.63-4.13) | 0.61 |
|  | A/A | 17 (15.9) | 19 (13.9) | 1.04 (0.49-2.20) |  |  | 26 (15.8) | 24 (14.1) | 1.09 (0.57-2.10) |  |  | 9 (15.5) | 5 (15.2) | 1.27 (0.36-4.48) |  |
|  |  |  |  |  |  |  |  |  |  |  |  |  |  |  |  |
| Allele | C | 141 (66.0) | 177 (65.0) | Ref. |  |  | 214 (65.0) | 222 (65.0) | Ref. |  |  | 73 (63.0) | 45 (68.0) | Ref. |  |
|  | A | 73 (34.0) | 97 (35.0) | 0.94 (0.65-1.37) | 0.841 |  | 116 (35.0) | 118 (35.0) | 1.04 (0.75-1.44) | 0.88 |  | 43 (37.0) | 32 (21.0) | 0.83 (0.44-1.56) | 0.63 |
|  |  |  |  |  |  |  |  |  |  |  |  |  |  |  |  |
| HWE |  | 0.054 | 0.57 |  |  |  | 0.06 | 0.24 |  |  |  | 0.58 | 0.23 |  |  |

OR: Odds Ratio, CI: Confidence Interval, n: number of individuals, HWE: Hardy Weinberg equilibrium, Ref.: Reference group.

* Only nonsmokers were analyzed.

† Adjusted for smoking habits.
